# Infants of Mothers with Cocaine Use: Review of Clinical and Medico-Legal Aspects

**DOI:** 10.3390/children9010067

**Published:** 2022-01-05

**Authors:** Clara Cestonaro, Lorenzo Menozzi, Claudio Terranova

**Affiliations:** Legal Medicine and Toxicology, Department of Cardiac, Thoracic, Vascular Sciences and Public Health, University of Padova, 35121 Padova, Italy; clara.cestonaro@hotmail.it (C.C.); lorenzo.m.menozzi@gmail.com (L.M.)

**Keywords:** maternal cocaine use, intrauterine cocaine exposure, birthweight

## Abstract

Illicit drug use is a global problem that also affects pregnant women. Substance use and alcohol abuse during pregnancy may have various harmful consequences for both mothers and foetuses. Intrauterine exposure to illicit substances can be investigated through maternal reports and toxicological tests on mothers’ and/or newborns’ samples. While the negative effects of alcohol and opioid use on pregnancy, the foetus, and/or newborn are well established, the effects of cocaine use remain controversial. We performed a review of the literature to evaluate the current state of knowledge of the effects of intrauterine cocaine exposure on newborns’ and children’s long-term development and to highlight possible implications for health professionals dealing with women who use cocaine during pregnancy. Although intrauterine cocaine exposure has been associated with reduced infant measurements, no specific amount of cocaine use exerting such effects has been determined, and no long-term effects have been confirmed. The evidence of cocaine use during pregnancy justifies a clinical and social takeover of the mother and newborn without assuming that there will certainly be long-term damage related to intrauterine cocaine exposure, but also considering other possible associated factors.

## 1. Introduction

Illicit drug use is a global health problem that affects people of all genders and age groups. In the United States, the National Center for Health Statistics reported that more than 11% of people aged 12 years and older used illicit drugs in the previous month [1]. The 2005 National Survey on Drug Use and Health estimated that there were 2.4 million frequent cocaine users in the United States. In addition to regular users, and excluding those in prisons, up to 4.6 million people reportedly used cocaine occasionally [2]. The 2010 National Survey on Drug Use and Health conducted by the National Institute on Drug Abuse reported illicit drug use rates among pregnant women of approximately 16% from 15 to 17 years of age, more than 7% from 18 to 25 years of age, and 1.9% from 26 to 44 years of age [3]. According to the World Health Organization’s guidelines for the identification and management of substance use and substance use disorders in pregnancy published in 2014, healthcare providers ‘should ask all pregnant women about their use of alcohol and other substances’ [4]. 

Further investigation into substance use among pregnant women could be conducted using various methods, including interviews and questionnaires, urine testing, and hair and meconium testing. Whereas urine testing allows the measurement of cocaine metabolites for 96–120 h after the last substance use, meconium and hair testing can detect non-recent use. Maternal hair segmentation could allow the assessment of cocaine use during different gestation periods. A fraction of hair at least 3 cm long is usually necessary for a three-month-period assessment. A 9-cm hair could enable the analysis of all three trimesters. However, shortcomings of the various methods cause difficulties in evaluating the prevalence of prenatal exposure to illicit substances. Moreover, confirmation procedures (whose forensic standard is gas chromatography/mass spectrometry) influence the accuracy of the detection of intrauterine substance exposure [5]. Knowledge of substance use and alcohol abuse during pregnancy is important due to the potentially harmful consequences for both women and their foetuses [6]. For example, alcohol abuse during pregnancy is associated with foetal alcohol syndrome, which is characterised by growth reduction, facial phenotypes, central nervous system injury, and evidence of intrauterine alcohol exposure [7]. Regarding opioids, a previous study identified neonatal abstinence syndrome (a withdrawal syndrome) in 30–80% of babies whose mothers used opioid agonist therapies [8].

Whereas the negative effects of alcohol and opioid abuse on pregnancy, the foetus, and/or newborn are well established, the effects of cocaine use remain controversial. Concurrent substance use, smoking, low socio-economic status, and a deficiency of acceptable prenatal care are factors that interfere with the correct interpretation of the consequences of maternal cocaine use on the foetus [9]. Ascertaining a causal relationship between cocaine use during pregnancy and its effects on foetal and postnatal growth is thus clinically and legally crucial. From a clinical point of view, a diagnosis of negative foetal and postnatal consequences would be associated with an effective treatment on a clinical and social/family level; from a legal point of view, a diagnosis could have consequences not only for parental responsibility, but also from civil and criminal perspectives. To evaluate the current state of knowledge of the consequences of prenatal cocaine exposure on newborns, as well as its long-term effects, we reviewed the relevant literature, highlighting potential implications for health professionals working with women who use cocaine during pregnancy.

## 2. Material and Methods

In June 2021, one of the authors (C.C.) conducted a review of the literature by searching MEDLINE/PubMed. To ensure that no studies would be missed, no temporal limits were set. The following search phrase was used: ‘intrauterine cocaine exposure and newborn effects’. C.C. also searched articles in PubMed using the search phrase ‘intrauterine cocaine exposure long-term effects’. The two searches returned 80 and 33 articles, respectively.

### 2.1. Inclusion Criteria

Studies on the relation between cocaine consumption during pregnancy and its effects on pregnancy, foetuses, newborns, and/or its long-term effects were to be included. No distinction in the method of cocaine consumption or in the gestation period (first, second or third trimester) was made.

### 2.2. Exclusion Criteria

Articles not fulfilling the above-mentioned criteria were excluded. Additionally, articles that were not written in English were excluded. Studies that mentioned animal models and/or concurrent substance use other than cocaine or used the general expression ‘drug use’ in their titles were also excluded. The study selection was performed by all three authors (C.C., C.T., and L.M.) based on titles and abstracts. All studies considered relevant by the authors were retrieved in full text.

**Figure 1:**
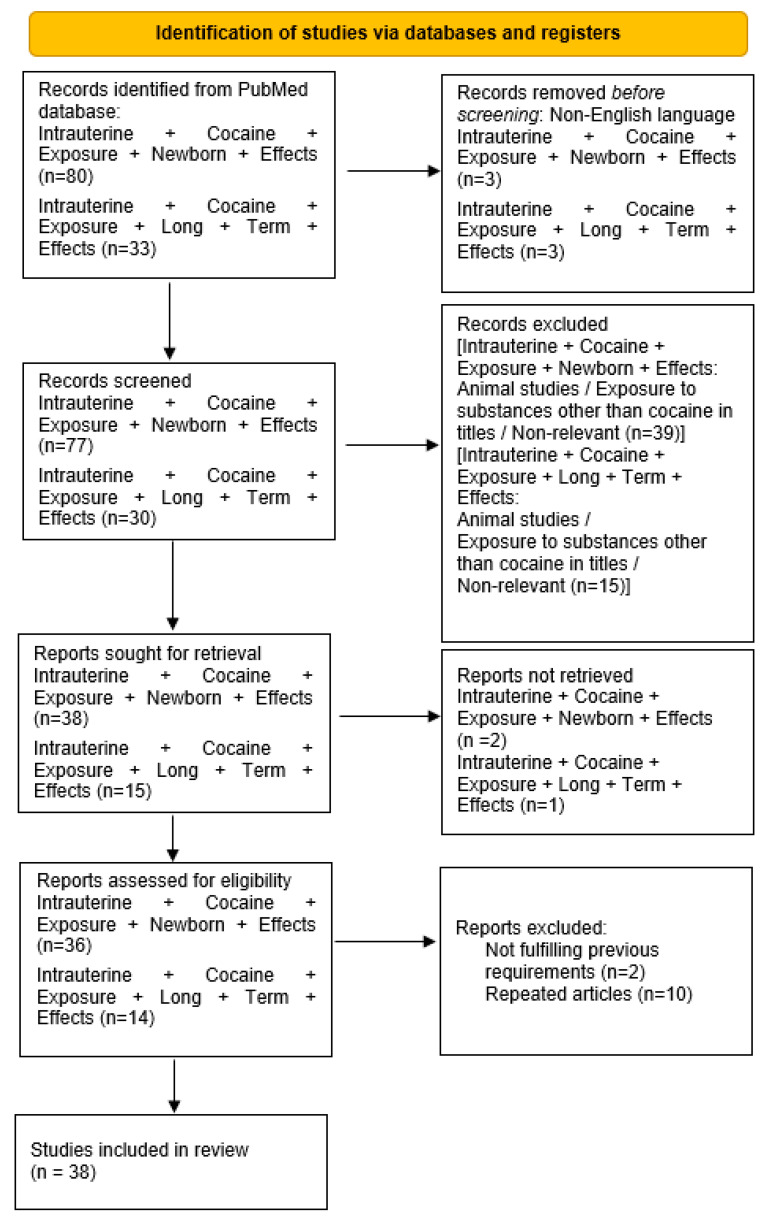
PRISMA flow chart [10].

A total of 36 articles were selected using the first group of keywords, and 14 articles were selected using the second group of keywords. A full-text reading was then performed. After excluding duplicate articles and those whose full texts did not fulfil the aforementioned criteria, 38 articles were finally reviewed. The numbers of articles included and excluded were registered and reported in a PRISMA flow chart (Figure 1).

## 3. Results

Table 1 summarises the populations, determination of cocaine exposure, and results of the reviewed studies. The results are discussed considering the specific system involved after cocaine exposure. The consequences of cocaine exposure were grouped into the following categories: birth timing, foetal consequences and infant measurements; neurological and neurobehavioural consequences; cardiac and respiratory consequences; other consequences; and long-term effects.

### 3.1. Birth Timing, Foetal Consequences and Infant Measurements

Sehgal et al. [11] studied a population of low-birthweight infants and observed that premature birth was the most significant adverse consequence of late-pregnancy cocaine use. The authors found that 66% of drug-exposed babies had birthweights of <1500 g compared with 50% of non-exposed babies and that newborns with birthweights of <1500 g were premature regardless of whether intrauterine growth was adequate. A review conducted by Singer et al. [12] found higher rates of preterm birth among cocaine-exposed infants, as well as a decreased gestational age in most studies. Similarly, Van de Bor et al. [13] observed a shorter gestational age in cocaine-exposed newborns, while Bandstra et al. [14] reported less maturity in terms of gestational age. A lower mean gestational age was also observed by Chasnoff et al. [15,16] and Cherukuri et al. [17], who examined crack users. Bandstra et al. [14] suggested that deficits in gestational age were related to cocaine exposure and not to lifestyle or background characteristics.

Regarding the principal consequences of cocaine exposure, most studies described effects on infant measurements, particularly foetal growth, birthweight, and head size. According to a review conducted by Singer et al. [12], most studies found that prenatal cocaine exposure led to reductions in birthweight, head circumference and length, and to an increased rate of low birthweight. Bateman et al. [18] reported a more-than-double risk of low birthweight and decreased length and head circumference among babies exposed to cocaine during pregnancy, especially when cocaine was used in ‘crack’ form or in combination with other drugs. To explain the reduction of foetal growth, the authors cited previous studies examining the modification of fat storage caused by cocaine, the possible alteration in nutrient transfer and foetal metabolism, and possible interference with maternal nutrition. In another study, Bateman et al. [19] studied babies of an estimated gestational age of >36 weeks with cocaine exposure identified through maternal radioimmunoassay of hair (RIAH). High levels of cocaine exposure in utero were considered those corresponding to RIAH of >81 ng/10 mg of hair. The findings showed that head circumference was disproportionately smaller in term and near-term newborns exposed to high levels of cocaine, suggesting that cocaine use inhibited foetal brain growth. As the study included only near-term babies admitted to a well-baby nursery, the authors noted that their findings applied only to healthy term or near-term newborns.

Bandstra et al. [14] studied a cohort of full-term newborns in a low socio-economic context and found that intrauterine cocaine exposure was related to decreased foetal growth. Conversely, they observed no decrease among newborns prenatally exposed to alcohol, tobacco, or marijuana without cocaine exposure. Moreover, their results contradicted the hypothesis of a direct effect of cocaine on head circumference.

Van de Bor et al. [13,20] also observed low birthweight among infants exposed to cocaine during pregnancy. However, in one of their studies, the authors indicated that few cocaine-exposed newborns had birthweights below the 10th percentile. Lower birthweight, length, and head circumference were noted by Lester et al. [21], Cherukuri et al. [17], Chasnoff et al. [15,16], Hadeed et al. [22], and Chiriboga et al. [23]. In particular, the results of Chiriboga et al. [23] based on cocaine exposure measurements by RIAH and considering newborns with a gestational age of >36 weeks suggested a dose–response relationship between increasing levels of chronic exposure to cocaine and foetal growth, with higher rates of symmetric intrauterine growth restriction in intrauterine cocaine–exposed newborns. Cocaine exposure in the last trimester was determined by RIAH. The exposure values ranged from 2 to 4457 ng/10 mg of hair. Cocaine exposure was stratified into no exposure, low exposure (2–66 ng/mg of hair), and high exposure (81–4457 ng/mg of hair).

Sallee et al. [24] reported a relationship between the level of cocaine exposure and head growth retardation. Based on interviews with mothers, maternal and infant urine testing, and benzoylecgonine (BE) analyses of babies’ hair, the authors found a disproportionately reduced head circumference percentile compared with the weight growth percentile among newborns with positive hair tests. Specifically, they found 28 neonates to be positive for BE (in the range of 716–5440 ng/g), and compared with 33 control infants, they described ‘a negative correlation approaching significance (…) between BE and head circumference’.

Richardson et al. [25] conducted interviews concerning cocaine use with a population of pregnant women with or without prenatal care and further studied newborn measurements, mostly within 24–48 h after delivery. Their results indicated a relationship between cocaine exposure during early pregnancy and symmetric growth retardation in women both receiving and not receiving prenatal care.

However, Chasnoff et al. [15] found that when cocaine was used only during the first trimester of pregnancy, intrauterine growth improved, and weight, head circumference, and length were not significantly smaller than those of non-exposed children. Surprisingly, and in contrast to other studies, a retrospective analysis of newborns with low birthweights and low gestational ages conducted by Hand et al. [26] found higher birthweights in newborns exposed to cocaine than in non-exposed controls. To account for these divergent findings, the authors hypothesised that their population of pregnant women consumed cocaine near the time of delivery, which had a lesser impact on birthweight than cocaine use throughout pregnancy.

Weathers et al. [27] reviewed clinical data with a particular focus on three- and six-month (±4 weeks) and 12-month (±6 weeks) baby visits. Despite a high percentage of children lost to follow-up (up to 50% for some parameters), the length and head circumference percentiles of cocaine-exposed babies increased to or above the 50th percentile at 12 months. A ‘catch-up’ phenomenon for length (and weight) was previously identified by Chasnoff, who had a lower percentage of loss to follow-up.

### 3.2. Neurological and Neurobehavioural Consequences

To investigate the effects of intrauterine cocaine exposure on the development of intraventricular haemorrhage in preterm babies, McLenan et al. [28] examined newborns in their first week of life using head ultrasound. A gestational age of ≤30 weeks was the most relevant factor related to the development of intraventricular haemorrhage. Apgar scores and pneumothorax were other associated factors, with pneumothorax showing the strongest association with the severity of intraventricular haemorrhage. However, intrauterine exposure did not affect the development and severity of intraventricular haemorrhage.

Van de Bor et al. [20] used two-dimensional/pulsed doppler cranial ultrasonography in a population of 20 full-term newborns prenatally exposed to cocaine. They observed increased mean peak systolic flow velocity, end diastolic flow velocity, and mean flow velocity in three cerebral arteries on the first day after delivery and an increased mean heart rate and arterial pressure compared with controls. The differences between the cases and controls disappeared the following day, and the higher cerebral blood flow velocity was presumed to reflect higher cerebral blood flow or a modification in cerebral vascular resistance.

Avants et al. [29] used magnetic resonance imaging in a selected population of adolescents and found structural effects on the dopaminergic system, possibly related to prenatal cocaine exposure. Focusing on adolescents with high levels of prenatal cocaine exposure, they observed that their left, right, and total caudate volumes were smaller than those of controls.

Chiriboga et al. [23] studied a population of newborns with a gestational age of >36 weeks and detected cocaine exposure using RIAH to investigate the neurological consequences of intrauterine cocaine exposure. They found increased rates of neurological abnormalities with intrauterine cocaine exposure and ‘a dose–response relationship between increasing levels of chronic cocaine exposure and neurologic function’. They also found that hypertonia was inversely related to being small for gestational age (SGA). Movement alterations suggested a direct effect on neural circuitry, particularly in the monoaminergic system. In another study, Chiriboga et al. [30] found higher rates of neurological abnormalities (in terms of tone and movement) in 14 prenatally cocaine-exposed infants than in controls.

Beltran et al. [31] also described transient dystonic reactions in a case series of four newborns whose mothers used cocaine and tobacco (as well as heroin in one case) during pregnancy. Le Blanc et al. [32] found short-duration neurological abnormalities (tremulousness, irritability, and muscular rigidity) in less than half of their population of children born to crack-abusing mothers.

Anecdotally, cocaine exposure has been associated with neurobehavioural abnormalities, such as sleep disorders, daytime crying, and attention deficit disorders. However, according to Chiriboga [33], to establish a causal link between foetal cocaine exposure and altered child behaviours, the influence of the mother’s psychopathology, and mother–child interactions on the child’s behaviours should be investigated.

Chasnoff et al. [15] performed behavioural assessments 12–72 h after the delivery of newborns whose mothers used or did not use cocaine during the first trimester or throughout pregnancy. They observed relevant impairments in children exposed to cocaine prenatally compared with non-exposed children in the areas of orientation, motor ability, and state regulation, as well as an increase in the number of abnormal reflexes. The authors hypothesised that cocaine exposure in the first trimester alone might be associated with a risk of neurobehavioural impairment and that the blockage of norepinephrine and dopamine reuptake could interfere with neuronal development.

Chasnoff [16] used the Neonatal Behavioural Assessment Scale to study full-term newborns at 12–72 h and one month of age and reported poor motor, orientation, and state regulation behaviours among cocaine-exposed babies. While the parameters improved at the one-month follow-up, the latter two measurements remained poorer than in non-exposed infants.

According to Bateman et al. [18], neurobehavioural symptoms, such as irritability, feeding difficulty, and tremors, were not frequent among cocaine-exposed babies who had not been concurrently exposed to other substances. Karmel et al. [34] studied healthy term newborns and found an absence of attention modulation when arousal was manipulated in prenatally cocaine-exposed children.

The relation between the acoustic characteristics of crying and exposure to cocaine was investigated by Lester et al. [21], who compared groups of babies with a gestational age of >36 weeks. The babies’ cries were recorded on the second day after delivery, and cocaine exposure was associated with a longer duration of crying, a higher fundamental frequency, and a higher and more variable first formant, independently of birthweight. Meanwhile, lower birthweight was associated with a longer cry latency, fewer overall cry utterances, a lower crying amplitude, and dysphonation or turbulence during crying.

In a prospective study conducted by Swanson et al. [35], who verified cocaine exposure using RIAH and evaluated four-month-old babies, prenatally cocaine-exposed babies had significantly higher full-scale Movement Assessment of Infants (a neuromotor assessment) total risk scores. The authors also observed relevant differences in volitional movement between babies exposed to cocaine during the third trimester and those exposed within the first two trimesters. The results suggested the presence of an adverse effect on motor outcomes beyond the neonatal period and deviations in upper extremity movements but no relevant differences in functional skills among cocaine-exposed children. The period of cocaine exposure also appeared to be an important factor influencing motor outcomes, suggesting that discontinuing cocaine use before the third trimester might reduce the risk of motor dysfunction. However, the authors emphasised that the long-term implications of tone and movement abnormalities are unknown.

### 3.3. Cardiac and Respiratory Consequences

The possible respiratory effects of intrauterine cocaine exposure among full-term and preterm newborns have also been investigated. Chasnoff et al. [36] performed pneumograms on two-week-old newborns delivered at or over 38 weeks of gestation. They found a higher frequency of cardiorespiratory pattern abnormalities among prenatally cocaine-exposed babies than among heroin/methadone-exposed children. Conversely, in a cohort of newborns with birthweights of 750–1500 g and a gestational age of <34 weeks, Hand et al. [26] found that cocaine-exposed children had a reduced need for surfactant treatment and initial intubation. The authors hypothesised that this was related to reduced uterine blood flow and higher catecholamine levels. Moreover, Beeram et al. [37] studied the medical records of newborns with birthweights of 500–1500 g and found no relevant differences in the incidence of respiratory distress syndrome between exposed and non-exposed low-birthweight newborns (with a mean weight of approximately 1000 g).

To evaluate the cardiac consequences of cocaine exposure, Mehta et al. conducted multiple studies [38,39,40,41] of newborns with birthweights of ≥1500 g. They found lower vagal tone and global heart rate variability (HRV) indexes in cocaine-exposed children than in controls. In particular, altered HRV was observed during the first three days of life. The reduction in both vagal tone and HRV was greater in babies heavily exposed to cocaine than in lightly exposed and non-exposed children. The decrease in HRV was attributed to effects on autonomic function or to a reduced sinus node response to extrinsic signals. Relevant differences in blood pressure at birth and at two to six months of age between cocaine-exposed babies and controls were not found. Moreover, a recovery of vagal tone was observed at two and six months of age in babies who had been lightly exposed to cocaine prenatally.

Mehta et al. also found more diastolic asynchronies in heavily in-utero-exposed children, which were hypothesised to be a consequence of uneven myocardial hypertrophy, localised interstitial fibrosis, and non-uniform loss of contractile elements. Moreover, the authors reported altered segmental filling of the left ventricle (within 48 h of birth) and a relationship between the frequency and severity of this alteration and the severity of cocaine exposure. The alteration seemed to disappear at two and six months of age, except for diastolic filling of the septal wall in the heavily cocaine-exposed group. According to the authors, these changes in the segmental filling fraction suggested regional involvement of the myocardium.

Van de Bor et al. [13] performed echocardiography on 15 full-term newborns in the first two days after delivery. On the first day, they observed a reduction in mean cardiac output and stroke volume and higher mean arterial blood pressure in cocaine-exposed newborns compared with non-exposed babies. However, the differences disappeared the following day.

### 3.4. Other Consequences

Few studies have described the conditions associated with intrauterine cocaine exposure, which have thus remained largely anecdotal. Mitra [42] reported a reduced foetal bladder cycle and urine output related to cocaine exposure. They measured the hourly urine production of foetuses between 24 and 38 weeks of gestation whose mothers had a history of cocaine abuse and positive urine tests and hypothesised an obstruction of the uretero-vesical junction secondary to detrusor contraction. Hoyme et al. [43] reported 10 cases of babies whose mothers abused cocaine during gestation. Nine babies exhibited congenital limb reduction and/or intestinal atresia or infarction. However, six of the children had been exposed to other substances as well.

The’ et al. [44] reported a case of intestinal perforation in a preterm infant whose mother had a history of alcohol and cocaine abuse until the day before delivery. The intestinal perforation was not related to necrotising enterocolitis or other gastrointestinal symptoms, and no blood chemistry changes were seen. An association between maternal cocaine use and necrotising enterocolitis was reported by Sehgal et al. [11], who studied a population of low-birthweight newborns. However, the authors were unable to determine whether the cause of this manifestation was vasoconstriction secondary to cocaine exposure or an epiphenomenon associated with asphyxia or intrauterine growth retardation.

### 3.5. Long-Term Effects

Regarding the duration of signs and symptoms, Chiriboga [33] reported that, except for inattentiveness, the effects of cocaine exposure on neurological and neuropsychological functions were self-limited and not permanent. Similarly, Doberczak et al. [45] described transient neurological effects. In their study, neurological abnormalities were most visible on the third day of life and improved even without pharmacological treatment in most cocaine-exposed children. Because of the disappearance of clinical signs along with the disappearance of cocaine metabolites, the authors suggested the presence of a neurotoxic action. Conversely, Karmel et al. [34], who examined babies at hospital discharge and at one month of age, concluded that behavioural patterns could not be considered transient, ‘as effects lasted at least until 1 month of age’.

According to a review conducted by Meyer et al. [46], while immediate and long-term effects of intrauterine exposure to cocaine have been found in animal models, a direct connection between maternal cocaine use and heart disease in adulthood has not been shown in human studies. This may be due to the absence of follow-ups of cocaine-exposed newborns in adulthood and the difficulty of distinguishing cocaine users from polydrug users.

Beeghly et al. [47] evaluated preadolescents from urban and low-income environments whose cocaine exposure was determined by combining biological markers and mothers’ self-reports. They found an association between intrauterine exposure and simple sustained auditory attention test results. However, the results were not dose-dependent, and other biological and environmental aspects were suspected to influence neuropsychological functioning.

According to Singer et al. [12], the difficulty in reaching conclusions about neurobehavioural effects is due to differences between sample populations (e.g., environment, prenatal care or use of other drugs). Moreover, the effects of cocaine on children’s behaviours are difficult to ascertain because of the well-known role of the environment in the development of at-risk children.

## 4. Discussion

Like a previous review [9] that highlighted the difficulty of obtaining accurate measurements of substance use during gestation, we found that the documentation of intrauterine cocaine exposure is often based on maternal history, mother and/or infant urine analysis, or both. Maternal history, which may be obtained through interviews and/or questionnaires, cannot be considered completely reliable. According to Giacoia [48], illicit substance use is frequently underreported, and Meyer et al. [46] argued that mothers may fear the effects of their revelations or the critical attitude of their physician. Regarding urine testing, which may be performed on mothers’ and/or children’ urine, Bateman et al. [18] stressed that urine tests of babies at birth provide information about cocaine exposure only within three to five days of delivery. Accordingly, Bateman noted that if women abstained from using cocaine several days before delivery, their newborns might be considered non-exposed. Furthermore, Meyer et al. [46] observed that, along with self-reporting cocaine use, urine testing may lead to a misidentification of users and inadequate differentiation between heavy and light users. The likelihood of misclassification [35] can be reduced by a combination of biological samples and history/interviews, which has also been shown to increase accuracy in identifying heavy and light exposure [40].

Although meconium testing enables the detection of cocaine exposure for a longer period [12,14,46,48], few of the reviewed studies included such testing. Moreover, while hair testing is more sensitive and makes it possible to trace back to the time of exposure, few of the reviewed studies investigated cocaine exposure using maternal RIAH. Sallee et al. [24] analysed newborns’ hair, noting that the possibility of external contamination was lower than when performing hair sampling months later. Finally, when information about tobacco, alcohol, and polydrug use is lacking, it is not possible to exclude their consumption and possible interfering effects during pregnancy.

Based on the information presented herein, this review confirmed the results of previous studies on prenatal cocaine exposure. The effects of cocaine exposure on infant measurements were demonstrated, although the long-term consequences remain unclear. Indeed, several studies with varying designs and populations have reported an association between intrauterine cocaine exposure and infant measurements, leading us to conclude that the principal consequences related to prenatal cocaine exposure are the negative effects on babies’ length, birthweight, and head circumference. These effects have been observed in populations of late-preterm (particularly a >36-week gestational age) and full-term newborns [13,14,19,20,21,23]. However, the effect on the birthweight of newborns with a low gestational age remains unclear. The association between maternal cocaine use during pregnancy and preterm birth and low birthweight was previously confirmed in a meta-analysis by Gouin et al. [9], who also found an association with SGA.

Whether cocaine exposure has specific neurological effects also remains unclear, as the observed effects on the central nervous system and neurological function vary and have mostly been investigated in isolated studies. An exception is tone alterations, which have been documented both in prospective studies [23,30] and in a case series [31]. Even fewer of the reviewed studies investigated the possible effects of cocaine exposure on cardiac and respiratory functions.

Specific long-term effects of cocaine exposure have not been established and have sometimes been refuted. Some studies describing specific manifestations at neonatal evaluation (e.g., altered vagal tone, cardiac output, and flow in cerebral arteries) reported that the differences between exposed and non-exposed babies disappeared within a few days or months. Moreover, some authors argued that conclusions about long-term effects were not possible because of the absence of human studies following up on cocaine-exposed babies into adulthood, as well as external factors, such as the environment, which may influence outcomes.

Because of the spreading epidemic of cocaine use [49], which affects diverse population groups, the results of our review have clinical and medico-legal implications. The clinical implications for paediatricians include the need to prepare for the eventuality of newborns with low birthweights and measurements and to refer women to facilities designed to accommodate such infants.

Regarding legal implications, concerning the United States, Kampschmidt reported that although few states support the criminal prosecution of women for their actions during pregnancy, ‘a large number of state prosecutors across the nation continue to bring cases despite the fact that the majority of cases are overturned on appeal’ [50]. Moreover, the ‘criminal justice system lacks the proper justification to punish women for their addictions that continue through pregnancy’ [50]. More recently, the American College of Obstetricians and Gynecologists (ACOG) [51] published a statement of policy concerning the ‘opposition to criminalization of individuals during pregnancy and the postpartum period’, in which 38 states were reported to have laws allowing foetuses to be considered victims of a crime. The statement concluded that the ‘criminalization of pregnant people for actions allegedly aimed at harming their foetus poses serious threats to people’s health and the health system itself’ [51].

In Italy, despite a law which rules the voluntary termination of pregnancy providing for the protection of human life from its beginning and a law on medically assisted procreation ensuring that the rights of the conceived are safeguarded, both the penal code and extra-code legislation have been considered insufficient for protecting the rights of the conceived [52]. However, based on the results of this review, the prosecution of mothers for injuries caused to foetuses during pregnancy in the case of cocaine consumption seems rebuttable. Furthermore, considering the uncertainty regarding the presence of neonatal lesions related to intrauterine cocaine exposure and—even more—in light of evidence suggesting that many of these lesions disappear within a short period, the appropriateness of reporting mothers or removing their newborns from them due to cocaine use is debatable.

It is therefore important to note that maternal and infant testing for substance use may give rise to consent issues. In this regard, the ACOG recommends that testing be performed only with the patient’s consent [53]. In Italy, recent legislation affirmed the right of every capable person to refuse a diagnostic assessment in part or in its entirety (except for cases stipulated by the law). For minors, consent to treatment is provided by their legal guardians. However, in the event of refusal of a treatment which a doctor considers appropriate and necessary, the decision is left to the judge [54].

Certainly, when the above-mentioned effects on infant growth are observed and intrauterine cocaine exposure is confirmed (not only through maternal history but also through analysis of biological markers), the diagnosis of a cocaine use disorder, as defined by the Diagnostic and Statistical Manual of Mental Disorders [55], must be considered. Knowing that the mother suffers from a substance use disorder is essential to allow social local services to take charge of the woman to treat her condition and of the newborn to ensure that growth occurs in a suitable environment and that adequate care is provided.

Indeed, even though the absence of clearly defined long-term effects makes it impossible to confirm that permanent lesions suffered by the child are due to maternal cocaine consumption during pregnancy, the protection of the child’s interests remains essential. Substance abuse during pregnancy may indeed exert various deleterious effects on mother–infant interactions. In particular, maternal misconduct has been associated with perturbations in oxytocin release [56]. Moreover, the first year after giving birth is stressful, and stress has been linked to the risk of relapse [57]. As the environment may influence the long-term outcomes of babies exposed to cocaine prenatally, when a history of maternal cocaine use during pregnancy emerges and a substance use disorder is diagnosed, particular attention should be paid to the environment in which the child will grow up. However, based on the results of this review, it cannot be assumed that intrauterine exposure to cocaine will cause long-term damage, and other possibly associated factors must also be considered.

## 5. Limitations and Scope for Further Research

Most of the reviewed studies were not recent, suggesting the need for further studies on the relationship between maternal cocaine use and its effects on pregnancy, the foetus, and the infant using rigorous scientific criteria. The need for scientific rigour is highlighted by the fact that most of the reviewed studies did not accurately establish the extent and duration of exposure to the substance. This factor, which was not used as an inclusion criterion to avoid excessively limiting the articles included, is a limitation of the study. Future studies could analyse mothers’ hair considering different hair segments. Hair segmentation could enable a focus on specific gestation periods. This kind of analysis, combined with examinations of mothers with the collection of data on other possible factors influencing newborns’ health, could help to identify more mothers using cocaine and clarify the relationship between cocaine, gestational age, and health consequences for the newborn.

## 6. Conclusions

The evidence of cocaine use during pregnancy justifies a clinical and social takeover of both the mother and the newborn without assuming that intrauterine exposure to cocaine will cause long-term damage, but also considering other associated factors. Although reductions in infant measurements have been associated with intrauterine cocaine exposure, no specific amount of cocaine consumption leading to the occurrence of such effects has been determined, and no long-term effects have been confirmed. Further studies are needed to investigate the long-term effects of cocaine exposure during pregnancy and to establish the exact relationship between the extent and duration of intrauterine cocaine exposure and its effects.

## Figures and Tables

**Table 1:** Populations, determination of cocaine exposure, and results of the reviewed studies.

Study	Study Design and Population	Prenatal Cocaine Exposure Assessment	Results
Sehgal et al. [11]	Case–control study 158 low-birthweight infants (500–2500 g) with a history of cocaine exposure admitted to neonatal intensive care units vs. 536 low-birthweight infants with no history of cocaine exposure	Drug-exposed infants identified either through a positive history or positive urine toxicology of the infant or mother	Low birthweight and increased incidence of necrotising enterocolitis in infants with intrauterine cocaine exposure
Singer et al. [12]	Review (methodological approach not described)	–	The way and degree to which foetal cocaine exposure leads to negative long-term effects on infant neurodevelopmental competence have not been established
Van de Bor et al. [13]	Case–control study 15 full-term newborns with a maternal history of cocaine use during pregnancy vs. 22 healthy full-term newborns admitted during the same period	Maternal history and mother’s and/or infant’s first urine samples after birth	On day 1 of life, infants exposed to cocaine had lower cardiac output, lower stroke volume, and higher arterial blood pressure. On day 2, cardiac output, stroke volume, and mean arterial blood pressure were similar
Bandstra et al. [14]	Case–control study 253 infants exposed prenatally to cocaine vs. 223 non-cocaine-exposed infants	Maternal interview and maternal and infant urine and meconium testing for cocaine metabolite (benzoylecgonine)	Growth deficits related to cocaine, symmetrical and partially mediated by gestational age
Chasnoff et al. [15]	Case–control study 75 cocaine-using women (Group 1: 23 women using cocaine in the first trimester; Group 2: 52 women using cocaine throughout pregnancy) vs. 40 women with no history or evidence of substance abuse	Maternal history and urine analysis using enzyme-multiplied immunoassay followed by gas chromatography/mass spectrometry	Group 2: increased rate of preterm delivery, low birthweight, intrauterine growth retardation; Group 1: rates of these complications similar to the drug-free group; mean birthweight, length and head circumference for term infants reduced in Group 2 infants; cocaine-exposed infants’ impairment of orientation, motor and state regulation behaviours measured using the Neonatal Behavioral Assessment Scale
Chasnoff et al. [16]	Case–control study 70 infants born to cocaine-using women vs. 70 drug-free infants	History and urine samples	High incidence of pregnancy complications in cocaine-addicted women; increased rate of intrauterine growth retardation, prematurity, microcephaly, and perinatal morbidity in infants born to cocaine-using women
Cherukuri et al. [17]	Case–control study 55 crack-using women vs. 55 parturients negative for drug use	Maternal and newborn urine testing	Higher risk of growth retardation and head circumference below the 10th percentile for gestational age in crack-exposed infants; transient abnormal neurobehavioural signs in 38% of crack-exposed infants
Bateman et al. [18]	Case–control study 361 cocaine-exposed infants vs. 387 infants not known to be exposed to cocaine	Maternal history or infant urine assay	Intrauterine cocaine exposure related to foetal growth retardation and shortened gestation
Bateman et al. [19]	Observational study 240 newborn infants (gestational age of >36 weeks) with exposure to cocaine. Cocaine exposure categorised into three levels: no exposure, low exposure and high exposure	Cocaine exposure assessed by maternal radioimmunoassay of hair (RIAH), additional maternal information from interviews and medical records, urine analysis of infants born to RIAH cocaine-positive mothers	Asymmetric intrauterine growth retardation, with head circumference disproportionately smaller than would be predicted from birthweight in infants with high intrauterine exposure to cocaine
Van de Bor et al. [20]	Case–control study 20 full-term newborn infants whose mothers had a history of cocaine use vs. 18 healthy full-term newborn infants whose mothers denied drug use	Maternal history, maternal and infant urine testing	On day 1 of life, cocaine-exposed infants had significantly higher peak systolic, end diastolic, and mean flow velocities in the pericallosal, internal carotid, and basilar arteries and mean arterial blood pressures. On day 2, cerebral flow velocities and mean arterial blood pressure were similar
Lester et al. [21]	Case–control study 80 cocaine-exposed infants vs. 80 controls	Data collected from the medical chart and based on urine tests (when available) or anamnesis	Lower birthweight, shorter length, and smaller head circumference in cases. Both direct and indirect effects (secondary to low birthweight) of cocaine on cries
Hadeed et al. [22]	Cohort study 56 newborn infants of mothers who used cocaine	Maternal history, maternal and infant urine samples	Growth retardation and microcephaly in newborns exposed to cocaine
Chiriboga et al. [23]	Case–control study 104 cocaine-exposed infants vs. 136 non-cocaine-exposed infants	Maternal radioimmunoassay of hair, additional maternal information from medical records, urine toxicology of a subset of infants and women	Dose–response relationship between cocaine exposure and adverse neonatal effects; higher rates of foetal head growth impairment and abnormalities of muscle tone, movements and posture in newborns with higher levels of prenatal cocaine exposure
Sallee et al. [24]	Cross-sectional study 34 infants born to mothers urine-positive for cocaine vs. 33 infants born to urine-negative mothers	Interview, maternal and urine testing, neonatal radioimmunoassay of hair	Head growth abnormalities associated with the levels of cocaine exposure
Richardson et al. [25]	Case–control study 295 women with prenatal care vs. 98 without prenatal care	Interview at the end of each trimester about use of cocaine, crack, alcohol, tobacco and other drugs	Growth retardation in cocaine/crack-exposed newborns
Hand et al. [26]	Retrospective cohort study 48 infants exposed to cocaine and 101 infants negative for drug exposure with birthweights of 750–1500 g and gestational age of <34 weeks	Maternal history and/or urine testing, infant urine testing	Short-term effects on the need for surfactant replacement therapy and initial intubation of exposed newborns with respiratory distress syndrome; no overall effect on the development of bronchopulmonary dysplasia
Weathers et al. [27]	Cohort study 137 infants with cocaine exposure during pregnancy	History or urine drug testing	Expected growth levels could be achieved by 1 year of age in cocaine-exposed children
McLenan et al. [28]	Observational study Cocaine-exposed preterm newborns examined by head ultrasound in their first week of life	Analysis of neonates’ first voided urine and/or maternal urine toxicology or history of drug use	No effect on the prevalence or severity of intraventricular haemorrhage in preterm infants exposed to cocaine
Avants et al. [29]	Case–control study 25 adolescents exposed to cocaine during pregnancy vs. 24 matched controls	Maternal urine testing at delivery	Dopaminergic system negatively affected by cocaine exposure during pregnancy
Chiriboga et al. [30]	Case–control study 14 prenatally cocaine-exposed infants vs. 16 unexposed infants	Maternal history, infant urine toxicology	Tone and movement abnormalities in newborn infants exposed to cocaine
Beltran et al. [31]	Case series 4 newborns positive for cocaine exposure	Maternal history and/or urine toxicology	Transient dystonic reactions initiated at 3 h to 3 months of age and continuing for months
Le Blanc et al. [32]	Observational study 38 children born to crack-using mothers	Maternal history, infant urine analysis	Mild and short-lived signs of central nervous system disfunction in less than half of the infants
Chiriboga [33]	Review (methodological approach not described)	–	Absence of evidence of detrimental long-term cocaine effects; no cognitive deficits related to foetal cocaine exposure, except as mediated through cocaine effects on head growth; abnormalities in neurological and neuropsychological function, self-limited and restricted to early infancy and childhood
Karmel et al. [34]	Observational study 180 infants with prenatal cocaine exposure	Maternal report, maternal or infant urine toxicology and/or meconium toxicology	Lack of appropriate arousal-modulated attention related to cocaine exposure in utero
Swanson et al. [35]	Case–control study 120 cocaine-exposed infants vs. 186 non-cocaine-exposed infants at 4 months	Maternal self-reports and (in most women) verification by maternal radioimmunoassay of hair	Adverse effects on infant motor development after the neonatal period related to the timing and duration of uterine cocaine exposure
Chasnoff et al. [36]	Case–control study 32 full-term and preterm 2-week-old cocaine-exposed infants vs. 18 heroin/methadone-exposed children 17 of 32 women used cocaine in the first trimester; 15 used cocaine throughout pregnancy	Urine screening	Higher incidence of cardiorespiratory pattern abnormalities in infants with intrauterine exposure to cocaine than in controls
Beeram et al. [37]	Case–control study 40 cocaine-exposed infants vs. 29 non-cocaine-exposed infants	Maternal or urine drug testing	Incidence of respiratory distress syndrome not influenced by intrauterine cocaine in very low-birthweight infants (<1500 g)
Mehta et al. [38]	Case–control study 68 infants with intrauterine cocaine exposure vs. 77 infants exposed to other drugs vs. 72 infants negative for drug exposure	Toxicological analysis of maternal urine, infant urine and meconium testing	Decreased heart rate variability associated with cocaine exposure
Mehta et al. [39]	Case–control study 71 cocaine-exposed infants (2–6 months old) vs. 89 newborns exposed to other drugs vs. 77 normal controls	Interview, questionnaire, toxicological analysis of maternal urine, infant urine and meconium testing	Lower heart rate variability in the first 72 h of life in cocaine-exposed infants with remission at 2–6 months of age; rebounding levels of vagal tone in infants exposed to light cocaine use; similar reduced response in heavy cocaine exposure
Mehta et al. [40]	Case–control study 82 newborns exposed to cocaine vs. 108 infants exposed to other drugs vs. 87 controls	Questionnaire, toxicological analysis of maternal urine, infant urine and meconium testing	Greater global and segmental fractional area changes and asynchrony during diastole in infants with intrauterine cocaine exposure
Mehta et al. [41]	Case–control study 56 2–6-month-old infants exposed to cocaine vs. 72 infants exposed to other drugs vs. 60 controls	Maternal self-report, maternal urine toxicological analysis, infant urine and meconium testing	At 2–6 months of age, infants exposed to cocaine recovered from left ventricular diastolic segmental alterations seen in the first 48 h of life; differences in heavily cocaine-exposed group
Mitra [42]	Case–control study Foetal hourly urine production and bladder cycle length in 36 pregnant women with cocaine abuse vs. 59 controls	Maternal history and urine drug screening for cocaine only on the day of the study	Reduced foetal urine output and bladder cycle in cases
Hoyme et al. [43]	Case series 10 infants with prenatal exposure to cocaine and other drugs	–	Congenital limb reduction and/or intestinal atresia or infarction in nine cases; limitations due to exposure to other substances
The’ et al. [44]	Case report	Maternal history and maternal and infant urine testing	Intestinal perforation in a preterm infant; mother with alcohol and cocaine abuse
Doberczak et al. [45]	Observational study 39 infants with intrauterine cocaine exposure	Maternal history and maternal/neonatal urine toxicologic assays	Transient and self-limited cocaine-related neonatal clinical neurological dysfunction
Meyer et al. [46]	Review (methodological approach not described)	–	Immediate and long-term cardiac consequences in animal model; human study incomplete but suggesting potential negative effects of cocaine exposure during development
Beeghly et al. [47]	Cohort study 137 preadolescents from low-income urban environments	History in medical record or neonatal or maternal urine toxicological screening, meconium assay	Association between cocaine exposure and simple sustained auditory attention test results; results not dose-dependent; other biological and environmental aspects suspected to influence neuropsychological functioning
Giacoia [48]	Review (methodological approach not described)	–	Microcephaly, growth retardation, brain infarcts, congenital malformations and withdrawal symptoms lasting for several weeks associated to cocaine exposure
